# Supplementary material for: The influence of pneumococcal positivity on clinical outcomes among patients hospitalized with COVID-19: A retrospective cohort study
Source: PLoS One. 2025 Aug 21;20(8):e0329474. doi: 10.1371/journal.pone.0329474 (PMC12370052; doi:10.1371/journal.pone.0329474)
Supplement: S1 Table — (DOCX) [file pone.0329474.s001.docx]

**S1 Table. Covariate balance before and after propensity score matching assessed by standardized mean differences (SMDs) comparing COVID-19 patients with and without pneumococcal positivity.**

| **Variables** | Pneumococcus (-), N=215  Mean/% | Pneumococcus (+), N=65  Mean/% | SMD (Before Matching) | SMD (After Matching) |
| --- | --- | --- | --- | --- |
| Sex, male (%) | 66.0 | 63.1 | 0.22 | 0.008 |
| Age, yrs | 73.16 | 76.14 | 0.061 | 0.014 |
| **Underlying Disease** |  |  |  |  |
| Hypertension (%) | 46.5 | 61.5 | 1.32 | 0.094 |
| Diabetes mellitus (%) | 35.3 | 44.6 | 0.19 | 0.062 |
| Chronic lung disease (%) | 34.0 | 32.3 | 0.036 | 0.032 |
| Chronic heart disease (%) | 26.0 | 38.5 | 0.267 | 0.124 |
| Chronic kidney disease (%) | 23.3 | 24.6 | 0.03 | 0.236 |
| Chronic liver disease (%) | 3.7 | 1.5 | 0.138 | 0.107 |
| Solid cancer (%) | 24.2 | 16.9 | 0.181 | 0.000 |
| Hematologic disease (%) | 17.7 | 4.6 | 0.416 | 0.180 |
| Cerebrovascular disease (%) | 23.3 | 29.2 | 0.134 | 0.033 |
| **Oxygen therapy** |  |  |  |  |
| High flow nasal oxygen therapy (%) | 35.3 | 50.8 | 0.313 | 0.000 |
| Non-invasive mechanical ventilation (%) | 0.5 | 3.1 | 0.196 | 0.251 |
| Invasive mechanical ventilation (%) | 20.0 | 16.9 | 0.08 | 0.041 |
| **Laboratory findings** |  |  |  |  |
| White blood cell, 10^3^/μl | 8.90 | 8.85 | 0.008 | 0.049 |
| Neutrophil, 10^3^/μl | 7.28 | 7.41 | 0.023 | 0.038 |
| Lymphocyte, 10^3^/μl | 1.02 | 0.98 | 0.036 | 0.142 |
| C-reactive protein, mg/dL | 12.01 | 12.13 | 0.013 | 0.131 |
| Procalcitonin, ng/dL | 4.00 | 13.98 | 0.318 | 0.179 |
| **Severity score** |  |  |  |  |
| CURB-65 | 2.03 | 2.45 | 0.335 | 0.092 |
| Pneumonia severity index | 130.9 | 141.4 | 0.233 | 0.09 |
| **Treatment** |  |  |  |  |
| Antiviral agent (%) | 85.1 | 90.8 | 0.175 | 0.045 |
| Corticosteroid (%) | 68.4 | 73.8 | 0.119 | 0.199 |
| Antibiotics (%) | 77.7 | 76.9 | 0.019 | 0.111 |

Abbreviations: SMD, Standardized mean difference
